# Immune checkpoint ligands expressed on mature high endothelial venules predict poor prognosis of NSCLC: have a relationship with CD8^+^ T lymphocytes infiltration

**DOI:** 10.3389/fimmu.2024.1302761

**Published:** 2024-02-08

**Authors:** Jing Luo, Xiuhuan Shi, Yumeng Liu, Jian Wang, Hao Wang, Xuena Yang, Qian Sun, Zhenzhen Hui, Feng Wei, Xiubao Ren, Hua Zhao

**Affiliations:** ^1^ Tianjin Medical University Cancer Institute and Hospital, National Clinical Research Center for Cancer, Tianjin, China; ^2^ Tianjin’s Clinical Research Center for Cancer, Tianjin, China; ^3^ Key Laboratory of Cancer Immunology and Biotherapy, Tianjin, China; ^4^ Department of Immunology, Tianjin Medical University Cancer Institute and Hospital, Tianjin, China; ^5^ Department of Medical Oncology, Affiliated Hospital of Inner Mongolia Medical University, Huhhot, China; ^6^ The Affiliated Jiangning Hospital of Nanjing Medical University, Nanjing, China; ^7^ Haihe Laboratory of Cell Ecosystem, Tianjin, China; ^8^ Department of Biotherapy, Tianjin Medical University Cancer Institute and Hospital, Tianjin, China

**Keywords:** lung cancer, immune checkpoint ligands, high endothelial venules, lymphocytes infiltration, tertiary lymphoid structures

## Abstract

**Background:**

An insufficient number of intratumoral CD8^+^ T lymphocytes is a major barrier to antitumor immunity and immunotherapy. High endothelial venules (HEVs) are the major sites through which lymphocytes enter tumors; however, the molecular mechanism through which HEVs mediate CD8^+^ T lymphocyte infiltration remains poorly understood.

**Methods:**

Forty-two patients with stage IIIA lung adenocarcinoma, who underwent surgery, were recruited. Multiplex immunohistochemical staining was conducted on tumor tissues to detect the immune checkpoint ligands (ICLs) expressed in the HEVs, blood vessels, and lymphatics. A new ICL score model was constructed to evaluate ligand expression. The relationship between ICL score, tumor-infiltrating CD8^+^ T cell frequency, and survival of patients was investigated.

**Results:**

Mature HEVs, but not blood vessels or lymphatics, mediated CD8^+^ T cell infiltration. However, the ICLs expressed on mature HEVs could negatively regulate CD8^+^ T cell entry into tertiary lymphoid structures (TLSs). In addition, according to the results obtained using our ICL_total_ score model, the expression of ICLs on HEVs was observed to be a predictor of both CD8^+^ T cell infiltration and survival, in which a high ICL_total_ score > 1 represent a weak CD8^+^ T cell infiltration and a high ICL_total_ score > 2 predicts poor survival.

**Conclusion:**

Using the ICL score model, we discovered that ICLs expressed on HEVs are indicative of CD8^+^ T cell subset infiltration in TLSs, as well as of patient survival with lung cancer.

## 1 Introduction

Since the early 2010s, immunotherapy has achieved monumental breakthroughs in cancer therapy and has revitalized the field of antitumor immunology (1). The clinical responses to immunotherapy have been strong, and the prognosis of patients has improved (1, 2). However, the efficacy of immunotherapy varies, and only specific subsets of patients benefit (3). Immune cell recruitment into the tumor microenvironment (TME) may be a critical factor in antitumor immunity and influence the clinical response and prognoses of cancer patients (4–6). Thus, obtaining an in-depth understanding of the immune infiltrates would be helpful in increasing clinical response and creating new therapeutic strategies for cancer prevention and treatment.

Lymphocyte delivery to tumors is essential for TME and antitumor immunotherapy (7–10). The high infiltration of T cells, especially CD8^+^ T subsets, into the TME is associated with a clinical response to immunotherapy in several types of cancer (11). Thus, further exploring the mechanisms governing immune cells delivery to tumors is critical. There are several ways in which blood vessels restrict lymphocyte extravasation (12); however, the mechanism of lymphocyte recruitment remains unclear. The chemokines C-X-C motif chemokine ligands 9 and 10 play important roles in T cell infiltration (13–16); however, the exact mechanisms of lymphocyte entry into the TME remain poorly defined.

High endothelial venules (HEVs), structurally and antigenically, are blood vessels that mediate lymphocyte delivery to the lymph nodes and other secondary lymphoid organs (4, 17, 18). HEVs play a critical role in the recruitment of lymphocytes as well as in immune surveillance of foreign invaders (bacteria or viruses) as they facilitate lymphocyte extravasation from the blood (19). The HEV system develops in the immune-stimulated lymph nodes during inflammation and is completely reconstructed in the tumor-draining lymph nodes. HEVs that exist extranodally are characteristically surrounded by lymphocytic forming lymph-nodes-like structures with distinct germinal centers and CD3^+^ T- and CD20^+^B-cell-rich areas, referred to as tertiary lymphoid structures (TLSs) (20). The development of TLSs in peripheral tissues is indicative of lymphoid neogenesis that occurs in response to long-term inflammation and they are very common to be found in many types of tumor tissues, according to most solid malignancies investigated thus far, the presence of mature TLSs is associated with a favorable prognosis, however, the degree of TLS maturation can affect immune function significantly, therefore, exploring the TLS maturation is crucial for immunotherapy (21). The periphery of TLSs is located by HEVs, which are the major sites through which lymphocytes enter tumors, govern the delivery of lymphocytes from the blood into the TME and providing specialized vasculature for TLS. HEVs are essential for the immune function and maturation of TLSs (22). As HEVs continue to mature, they mature becoming peripheral-node-addressin (PNAd) expressing HEVs (20), the presence of PNAd is regarded as a sign of maturity. The presence of TLSs with HEVs in human tumors are drawing increased attention owing to the therapeutic potential (22, 23). Both TLSs and HEVs play essential roles in regulating the recruitment of lymphocytes into the TME; however, there are many problems remain to be addressed. As mature HEVs, they have different capacities for delivery T cells, this may contribute to the differences in therapeutical response of immunotherapy between individuals, but the molecular and functional mechanisms remain poorly defined.

In this study, we found the expression of immune checkpoint ligands (ICLs) on mature HEVs negatively regulate the infiltration of CD8^+^ T cells into the TLSs. In addition, the presence of ICLs on HEVs may be a predictor of CD8^+^ T cells infiltrating the TLSs as well as of patient survival. This study provides a novel understanding of the HEVs governing lymphocyte delivery into the TLSs, and an index for predicting patient prognosis based on the total ICL (ICL_total_) score model developed in the study.

## 2 Materials and methods

### 2.1 Patients and tumor samples

A total of 49 patients with stage IIIA primary lung adenocarcinoma who had underwent surgery at our hospital between January 2015 and May 2016 were recruited, tumor tissues were collected, and immunohistochemical staining was performed to detect the TLS. All patients were classified into grades 1–3 based on the maturity level of TLSs as reported in our previous work (24), TLSs in patients of grade 3 were considered the most mature, whereas TLSs in patients of grade 1 were considered naïve. Given that naïve TLSs are PNAd-negative, we exclude patients with TLS maturity grade 1. Only patients with TLS maturity grade 2-3 were recruited. Every patient received 4 cycles of platinum doublet chemotherapy with pemetrexed after surgery, at a frequency of 21 days per cycle. No other treatment was administered. The eligibility criteria were as follows: (I) complete clinical data; (II) age 18–75 years; (III) pathologically confirmed lung adenocarcinoma; (IV) TLS maturity grade 2-3; (V) clinical stage IIIA; (VI) surgical resection of R0; and (VII) received standardized postoperative treatment. Finally, a total of 42 patients are recruited, the clinical characteristics of these patients are provided in **Table 1**.

**Table 1:** Clinical characteristics in patients with stage IIIA LUAD (n=42).

Characters		N (%)
Age, y		
	≥ 60	18 (42.9)
	< 60	24 (57.1)
Gender		
	Male	18 (42.9)
	Female	24 (57.1)
Smoking		
	Yes	17 (40.5)
	No	25 (59.5)
KPS		
	≥ 60	40 (95)
	< 60	2 (5)
T stage		
	T1	26 (61.9)
	T2	13 (31)
	T3	3 (7.1)
N stage	N1	3 (7.1)
	N2	39 (92.9)
Micropapillary		
	Yes	18 (42.9)
	No	24 (57.1)
Tumor volume		
	≥10 cm^3^	15 (35.7)
	<10 cm^3^	27 (64.3)
Type of resection		
	Lobectomy	30 (71.4)
	sleeve lobectomy	12 (28.6)

y, year; KPS, Karnofsky performance status.

### 2.2 Multiplex immunohistochemistry and multispectral analysis

Tumor tissues were collected from 42 patients, and multiplex immunohistochemical staining was conducted using a PerkinElmer Opal 7-color Technology Kit, according to the manufacturer’s instructions. Five staining panels were designed and applied to each panel to target different markers. HEVs were defined as PNAd^+^, while blood vessels and lymphatics were defined as CD31^+^ or PDPN^+^. Details of the panels and the relative antibodies are provided in **Table 2**. Each stained slide was visualized and quantified using a TissueFAXS Spectra System and StrataQuest analysis (TissueGnostics), according to previously described methods (25). The immune checkpoint molecule expression analysis is performed using the contextual tissue cytometry image analysis. Multispectral images were scanned using a 20× objective lens, and 10 fields were randomly selected for each slide. We collected all TLSs from all tumor sections; in total, 821 fields were collected, including 692 TLSs.

**Table 2:** Details of antibodies used in the multiplex immunohistochemistry staining.

Antibodies	Source	Identifier	Dilution
Recombinant Anti-CD3 antibody	abcam	ab135372	1:400
Anti-CD4 antibody	abcam	ab133616	1:400
Anti-CD8 alpha antibody	abcam	ab101500	1:1000
Anti-CD20 antibody	abcam	ab9475	1:600
Anti-LAG-3 antibody	abcam	ab180187	1:500
Anti-TIM 3 antibody	abcam	ab241332	1:250
Anti-PD1 antibody	abcam	ab137132	1:100
Peripheral Node Addressin Antibody	Novus Biologicals	NB100-77673	1:100
Anti-CD31 antibody	abcam	ab9498	1:500
Anti-Podoplanin antibody	abcam	ab236529	1:200
Anti-galectin 9/Gal-9 antibody	abcam	ab153673	1:100
Anti-HMGB1 antibody	abcam	ab79823	1:1200
Anti-CEACAM1 antibody	abcam	ab108397	1:50
Anti-LSECtin antibody	abcam	ab181196	1:200
Anti-FGL1 antibody	abcam	ab275091	1:400
Anti-Galectin 3 antibody	abcam	ab76245	1:1200
Anti-MHC Class II antibody	abcam	ab55152	1:800
PD-L1 (CD274) Recombinant Rabbit Monoclonal Antibody	Invitrogen	MA5-27896	1:300

The spatial distribution between the cells was quantified using the Dilate algorithm, which defines the cell sociology for each selected area. Finally, we developed the corresponding algorithms based on the analysis requirements, and for each channel, we applied a unified algorithm and threshold to all samples to standardize the expression and fluorescence levels of each marker. Based on previous studies (26, 27), spatial distance was analyzed for radius (r) = 30 μm, representing the proximity distance as the average number of cells distributed from the nuclear center of any reference cell.

### 2.3 Statistical analysis

Analyses of the results was conducted using SPSS (version 22.0). Kaplan–Meier analyses were used to analyze and compare differences in survival. The clinical characteristics of the patients were analyzed using the chi-square test. Disease-free survival (DFS) was defined as the date of tumor recurrence or diagnosis of metastasis. Statistical tests were two-sided, and a *P*-value < 0.05 was considered statistically significant.

## 3 Results

### 3.1 Frequency of CD8^+^ T cells is high in mature TLSs

**Figure 1:**
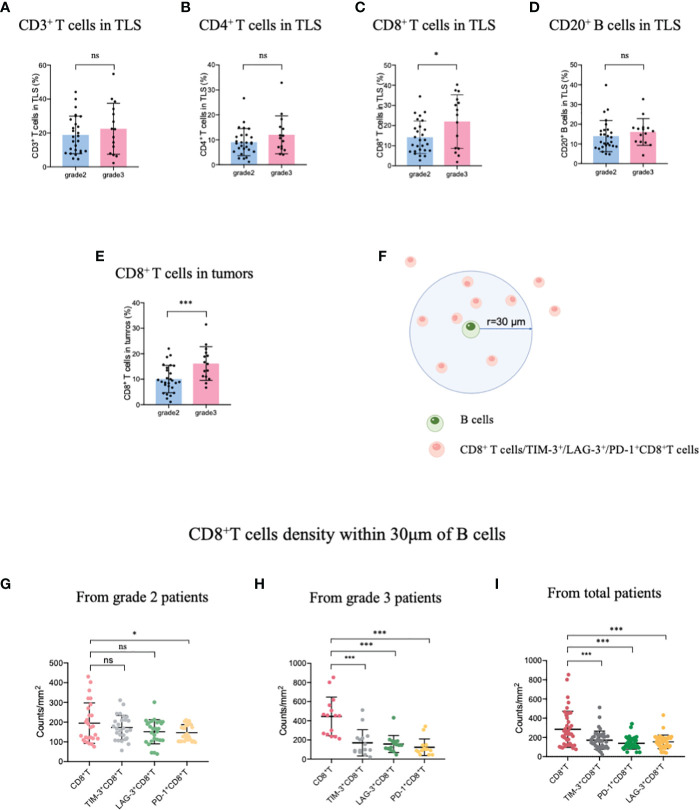
Mature HEVs positively affect CD8^+^ T cell entry into TLSs. The infiltration of **(A)** CD3^+^ T-, **(B)** CD4^+^ T-, **(C)** CD8^+^ T-, and **(D)** CD20^+^ B-cells into TLSs of patients with different TLS maturity grades. The infiltration of **(E)** CD8^+^ T cells into tumors of patients with different TLS maturity grades. **(F)** Illustration of spatial analysis methodology. Densities of cells of interest within a certain radius (30 μm) to a reference cell were calculated. Densities of CD8^+^ T cells and exhausted CD8^+^ T cells within 30 μm of CD20^+^ B-cells in patients with **(G)** grade 2, **(H)** grade 3, and **(I)** in all patients. HEVs, high endothelial venules. TLSs, tertiary lymphoid structures. Data are presented as mean ± SD. *P < 0.05; *** *P* < 0.01; ns, not significant according to unpaired two-tailed Student’s t-test.

Patients with TLS maturity grade 2-3 were recruited in this study, the infiltration of the immune population in the TLSs was evaluated for patients with different TLS maturity grades, and the data indicated that infiltration did not differ among CD3^+^ T, CD4^+^ T, and CD20^+^ B cells, representative images showing T lymphocytes and TLS (defined by B cells) were provided in **Supplementary Figure S1**. The proportion of only CD8^+^ T cells was higher in patients with grade 3 TLS maturity than in those with grade 2 TLS maturity (**Figures 1A–D**). Next, we evaluated the infiltration of CD8^+^ T cells in whole tumor samples, it was observed that the percentage of CD8^+^ T cells was higher in patients with grade 3 TLS maturity than in those with grade 2 TLS maturity (**Figure 1E**). These findings suggested that higher the maturity of the TLSs, higher is the number of CD8^+^ T cells that infiltrate the TLS or the TME. Based on these results, we analyzed the spatial relationship between CD8^+^ T/exhausted CD8^+^ T subsets and the TLSs. The spatial distributions of exhausted CD8^+^ T cells (TIM-3^+^/LAG-3^+^/PD-1^+^ CD8^+^ T cells) (28) and TLSs (CD20^+^ B cells) were explored. The bivariate K(r) function (29) was used to describe the spatial distribution of the two cell phenotypes. The radius used (30 μm) is generally considered ideal for calculating the spatial relationship between two cell populations (**Figure 1F**). In patients with grade 2 TLS maturity, compared with normal CD8^+^ T cells, only PD-1^+^ CD8^+^ T exhausted cells were found a few reductions around B cells, while other exhausted T cells remained unchanged. However, in patients with grade 3 TLS maturity, a marked decrease in the levels of all exhausted CD8^+^ T cells around B-cells compared with those of CD8^+^ T cells was observed, suggesting that exhausted CD8^+^ T cells were far from mature TLSs (**Figures 1G–I**). Altogether, these results suggest that the maturity of TLSs is positively correlated with CD8^+^ T lymphocytes delivery into TLSs.

### 3.2 Checkpoint ligands expressed in mature HEVs are negatively related to CD8^+^ T cells infiltrating the TLSs

**Figure 2:**
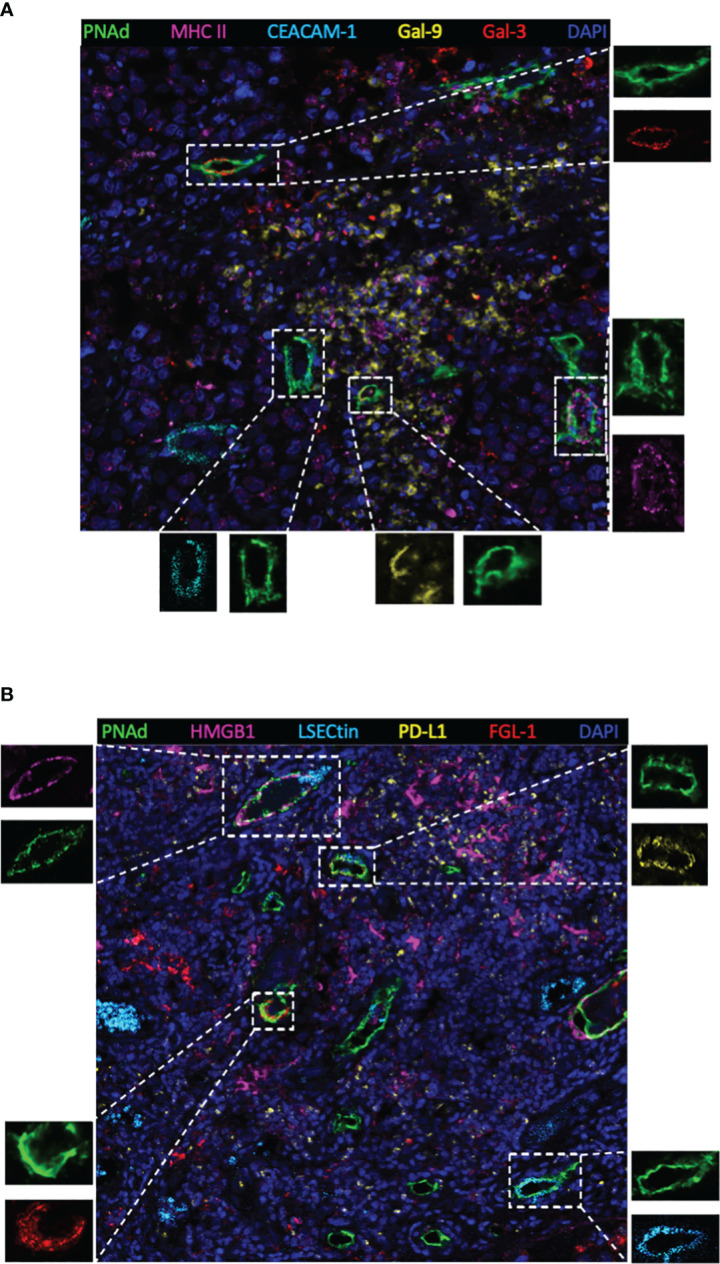
Representative multiplex immunohistochemistry staining image (20×) showing ligands expressed on mature HEVs. **(A)** Ligands of MHC II, CEACAM-1, Gal-9 and Gal-3 expressed on mature HEVs. **(B)** Ligands of HMGB1, LSECtin, PD-L1, FGL-1 expressed on mature HEVs.

**Figure 3:**
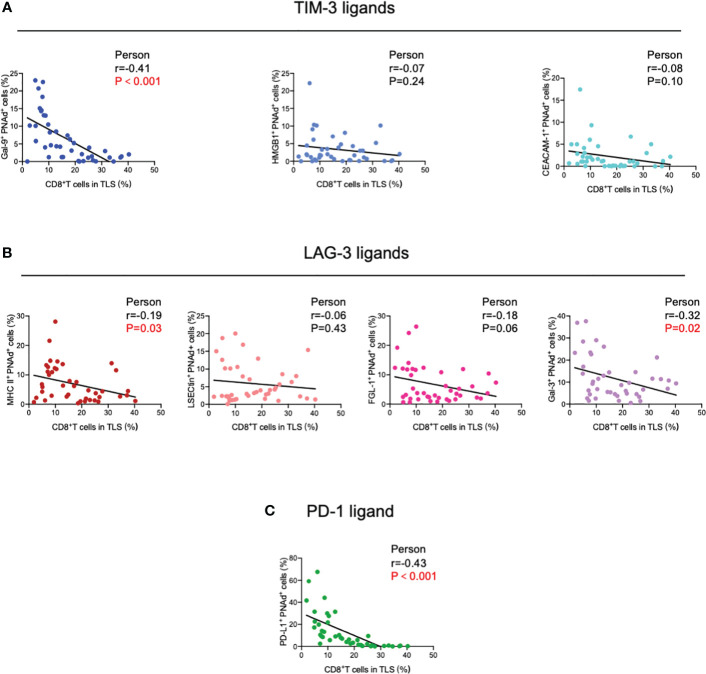
Correlation between **(A)** TIM-3, **(B)** LAG-3, and **(C)** PD-1 ligands expressed on mature HEVs and TLS-infiltrating CD8^+^ T cell frequency. HEVs, high endothelial venules. TLSs, tertiary lymphoid structures. Data are presented as mean ± SD. * *P* < 0.05; *** *P* < 0.01; ns, not significant according to unpaired two-tailed Student’s t-test.

Next, TIM-3, LAG-3, and PD-1 were chosen as they were the most common immune checkpoints and are very critical for immune regulation (30, 31); we then analyzed their ligands expressed on mature HEVs (PNAd was used as the marker of mature HEVs), blood vessels, and lymphatics, representative images were provided in **Figures 2A, B** and **Supplementary Figures S2**, **S3**. A negative relationship was found between the ligands expressed on mature HEVs and the TLS-infiltrating CD8^+^ T cell frequency, especially for Gal-9 (ligand of TIM-3), MHC II, Gal-3 (ligand of LAG-3), and PD-L1 (ligand of PD-1) (*P* < 0.05) (**Figures 3A–C**). When ligands were highly expressed in mature HEVs, the frequency of TLS-infiltrating CD8^+^ T cells decreased. Notably, these results were observed only for mature HEVs; ligands expressed on blood vessels or lymphatics did not impact TLS-infiltrating CD8^+^ T cell frequency (**Supplementary Figures S4A–C** and **Supplementary Figures S5A–C**). These data indicate the importance of the checkpoint ligands expressed on mature HEVs, which may negatively affect CD8^+^ T cell entry into tumors. Additionally, not every ligand expressed on HEVs has the ability to substantially affect tumor-infiltrating CD8^+^ T cells; only Gal-9, MHC II, Gal-3, and PD-L1 play important roles in mediating CD8^+^ T cell subsets’ entry into the TLSs. To further confirm the importance of ICL expressed on mature HEVs, we analyzed the expression of all ICL in the TME and found that patients with high ICL score have a relatively higher expression of ICL in TME than those with low ICL score, but the differences are not statistical (**Supplementary Figure S6A**). We then investigated the relationship between the 4 important checkpoint ligands (Gal-9, MHCII, Gal-3 and PD-L1) expressed on HEVs and those expressed in TME. Results showed that for the 3 important ligands (MHCII, Gal-3 and PD-L1), correlations were found between their expression levels on HEVs and in TME (**Supplementary Figure S6B**). Nevertheless, the ICL expressed on other sites of tumors except HEVs might have minimal effects on CD8^+^T cell delivery (**Supplementary Figure S6C**).

**Figure 4:**
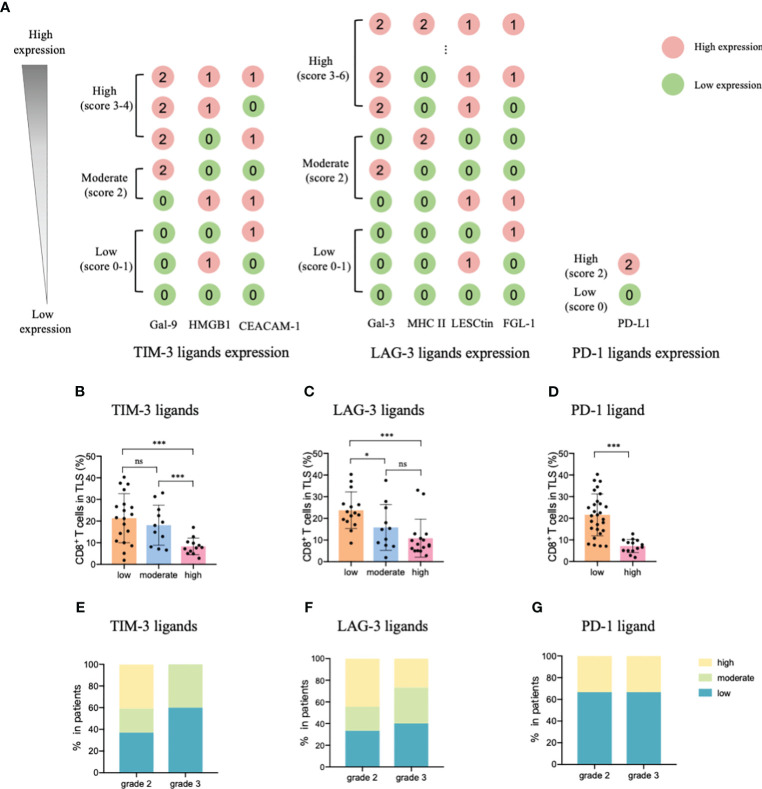
TIM-3, LAG-3, and PD-1 ligands expressed on mature HEVs negatively affected the tumor-infiltrating CD8^+^ T cell frequency. **(A)** The scoring pattern of the developed ICL score model used to calculate the expression levels of three checkpoint ligands. The effect of **(B)** TIM-3, **(C)** LAG-3, and **(D)** PD-1 ligand expression levels on mature HEVs on tumor-infiltrating CD8^+^ T cell frequency. Comparison of **(E)** TIM-3, **(F)** LAG-3, and **(G)** PD-1 ligand expression levels among patients with different TLS maturity grades. HEVs, high endothelial venules. ICL, immune checkpoint ligand. TLSs, tertiary lymphoid structures. Data are presented as mean ± SD. * *P* < 0.05; *** *P* < 0.01; ns, not significant according to unpaired two-tailed Student’s t-test.

Considering these results, we constructed an ICL score model to convert the eight ligands into three indices and evaluated the integrative ligand expression level of each immune checkpoint. First, the median value of the expression of each ligand was calculated. When a ligand expression value was larger than its median value, the ligand expression status was defined as “high expression;” otherwise, the status was defined as “low expression.” Next, considering the differences in the impacts of each ligand’s expression on tumor-infiltrating CD8^+^ T frequency, for the four important ligands (Gal-9, MHC II, Gal-3 and PD-L1), the item is scored as “2” when any one of them is defined as “highly expression”; otherwise, it is scored as “0”. Although the expression of the other four ligands (HMGB1, CEACAM-1, LSECtin, and FGL-1) did not considerably affect the frequency of tumor-infiltrating CD8^+^ T cells, the negative relationship was consistent for the four important ligands. Thus, when a patient had a high expression of any of the less important four ligands, the patient was assigned an score for the item of “1;” otherwise, the score was “0”. The ICL score for each checkpoint was the sum of its ligands scores. The higher the score, the higher the checkpoint ligands expression in the HEVs. We classified patients into three categories (low, moderate, and high checkpoint ligand expression) by their total checkpoint ICL score. The details of the scoring model are shown in **Figure 4A**. We investigated the frequency of TLS-infiltrating CD8^+^ T cells for various checkpoint ligand levels. We found that higher expression of TIM-3, LAG-3, or PD-1 ligand on the HEVs, representing a lower percentage of TLS-infiltrating CD8^+^ T cells; and, lower the ligand expression, higher was the CD8^+^ T cell infiltration into the TLSs (**Figures 4B–D**). Differences were statistically significant. According to the maturity level of the TLSs, patients were classified into one of the three grades, as described above (24); the expression of checkpoint ligands for the patients classified as different grades was measured. We found that for patients with grade 3 TLS maturity, who had the most mature TLSs, the expression of checkpoint ligands on HEVs was lower; and for patients with grade 2 TLS maturity, who had less mature, the expression of checkpoint ligands on HEVs was higher (**Figures 4E–G**). Based on these data, we speculated that the less mature HEVs express a higher number of checkpoint ligands and potentially decrease CD8^+^ T cell infiltration in TLS, whereas the mature HEVs express fewer ligands, which may increase CD8^+^ T cell infiltration.

**Figure 5:**
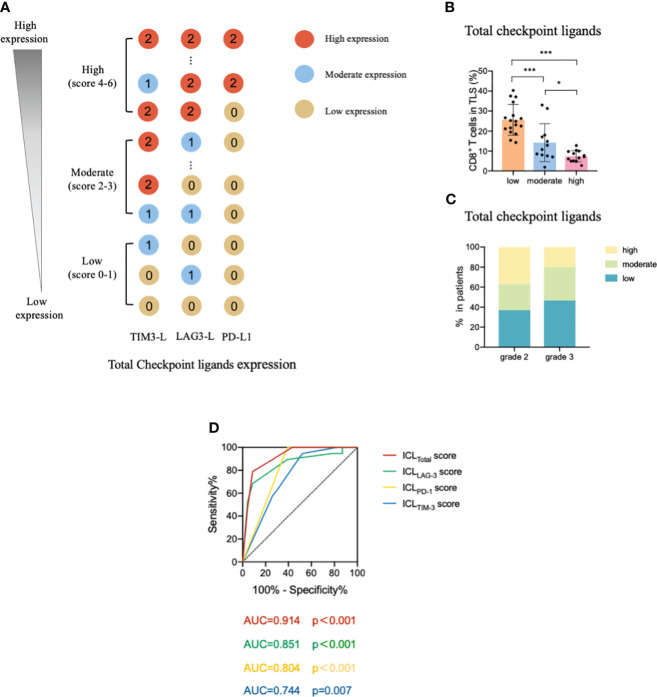
Levels of total ligands expressed on mature HEVs have a negative effect on the TLS-infiltrating CD8^+^ T cell frequency. **(A)** The scoring pattern of the ICL score model used to calculate the total ligand expression level. **(B)** The effect of total ligand expression levels on mature HEVs on the TLS-infiltrating CD8^+^ T cell frequency. **(C)** Comparison of total ligand expression levels for patients with different TLS maturity grades. **(D)** The ROC curve between TLS-infiltrating CD8^+^ T cell frequency and ICL scores. HEVs, high endothelial venules. TLSs, tertiary lymphoid structures. ROC, receiver operating characteristic. ICL, immune checkpoint ligand. Data are presented as means ± SD. * *P* < 0.05; *** *P* < 0.01; ns, not significant according to unpaired two-tailed Student’s t-test.

To comprehensively evaluate the checkpoint ligands expressed on mature HEVs, we used the developed ICL score model to convert the three checkpoint ligand expression indicators (TIM-3, LAG-3, and PD-1) into one index representing total checkpoint ligand expression levels. The details are shown in **Figure 5A**. According to the ICL_total_ scores, we classified patients into three classifications: high, moderate, and low total ligand expression. The effect of total ligand expression on the frequency of TLS-infiltrating CD8^+^ T cells was investigated, total tumor-infiltrating CD8^+^ T cells and the exhausted CD8^+^T cells (TIM-3^+^/LAG-3^+^/PD-1^+^/PD-1^+^TIM-3^+^CD8^+^T cells) were analyzed, results showed that a higher total checkpoint ligand expression in HEVs represented a lower percentage of TLS-infiltrating CD8^+^ T cells, whereas a lower total ligand expression indicated increased CD8^+^ T cell infiltration (**Figure 5B**), with the differences being statistically significant. The significant associations between the ligands expression levels and clinicopathological parameters were not observed (**Table 3**). And for the exhausted CD8^+^T cells, 2 kinds of exhausted CD8^+^T cells (PD-1^+^/PD-1^+^TIM-3^+^CD8^+^T cells) were observed an increase in high ICL expression patients (**Supplementary Figure S7**). This may due to the ICL high expression patients has a weak ability to deliver CD8^+^T cells into tumors, and those CD8^+^T cells have to fight against more tumor cells which causing exhaustion. Furthermore, for patients with grade 2 TLSs maturity, the expression of checkpoint ligands was higher in HEVs, whereas for patients with grade 3 TLS maturity, the expression was lower in HEVs (**Figure 5C**). These results provide evidence that less mature HEVs have higher checkpoint ligand expression, which may negatively affect the CD8^+^ T cells delivery into TLSs. As the HEVs mature, the ligand expression decreases, and CD8^+^ T cells can more easily infiltrate the HEVs, as we speculated.

**Table 3:** Associations between ICL expression levels and important clinicopathological parameters.

		Total ICL expression level			P value
		High	Moderate	Low	
Gender					0.313
	Male	7	3	8	
	Female	6	9	9	
Age, y					0.475
	≥ 60	4	5	9	
	< 60	9	7	8	
Smoking					0.395
	Yes	4	4	9	
	No	9	8	8	
Micropapillary					0.433
	Yes	5	7	6	
	No	8	5	11	
Tumor volume					0.331
	≥10 cm^3^	5	6	4	
	<10 cm^3^	6	8	13	

y, year; ICL, immune checkpoint ligand.

To quantify the negative effect of the ICLs of HEVs on the delivery of CD8^+^ T cells into TLSs, receiver operating characteristic (ROC) analysis was conducted to assess the accuracy of ICL scores in predicting TLS-infiltrating CD8^+^ T cell frequency (**Figure 5D**). Patients with a TLS-infiltrating CD8^+^ T cell frequency lower than the median value were considered to have “weak CD8^+^ T cell infiltration;” otherwise, patients were considered “strong CD8^+^ T cell infiltration.” The data showed that all four indices had large area under the curve (AUC) values, especially the ICL_total_ score indicator, which had an AUC value of 0.914 (*P* < 0.001), indicating that the ICL_total_ score could accurately predict TLS-infiltrating CD8^+^ T cell frequency. The cutoff value for the ICL_total_ score was evaluated, and a value of one was identified as the cutoff value that conferred 91.3% sensitivity and 78.9% specificity, indicating that an ICL_total_ score > 1 leads to weak CD8^+^ T cell infiltration (*P* < 0.001).

### 3.3 High ICL expression on HEVs predicts survival loss

**Figure 6:**
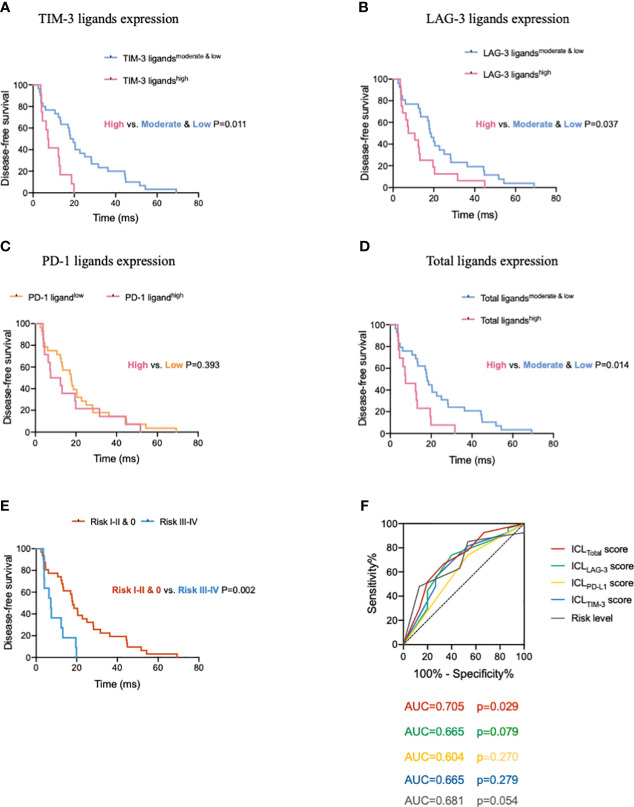
High expression of immune checkpoint ligands on mature HEVs predicts poor survival in patients with tumors. Comparison of DFS for patients with different **(A)** TIM-3, **(B)** LAG-3, **(C)** PD-1, and **(D)** total ligand expression levels. **(E)** Comparison of DFS of patients with different risk levels. **(F)** The ROC curve between DFS and the immune checkpoint ligands scores as well as the risk levels. HEVs, high endothelial venules. DFS, disease-free survival. ROC, receiver operating characteristic. Data are presented as means ± SD.

After confirming the effects of HEV-expressed ICLs on tumor-infiltrating CD8^+^ T cells, we analyzed the prognostic predictive ability of ICL levels in patients. The median age of the entire population (57.1% women, 42.9% men) was 57 years, and 95% of the patients had a Karnofsky performance status score ≥ 80. All patients had stage IIIA disease; the median DFS was 15.3 months for the whole population. The results of the survival analysis showed that the survival of patients with higher ICL expression was substantially worse (**Figures 6A–D**). The median DFS of patients for the low, moderate, and high TIM-3 ligand expression groups was 22.8 months (95% confidence interval (CI), 12.8–32.7), 17.0 months (95% CI, 9.4–24.7), and 7.2 months (95% CI, 5.2–9.1), respectively. The difference between the moderate & low and high TIM-3 ligand expression levels was significant (*P* = 0.011). The median DFS of patients for the low, moderate, and high LAG-3 ligand expression groups was 20.5 months (95% CI, 13.9–27.1), 18.1 months (95% CI, 12.4–23.7), and 7.4 months (95% CI, 0.5–14.4), respectively. The difference between the moderate & low and high LAG-3 ligand expression groups was significant (*P* = 0.037). Additionally, the median DFS of the patients for the low and high PD-1 ligand expression groups was 17.6 months (95% CI, 11.6–23.5) and 7.4 months (95% CI, 0–17.0), respectively. We found no significant difference between the low and high PD-1 ligand expression groups (*P* = 0.393). Finally, we analyzed the total ligand expression; the results indicated that the median DFS of patients for the total low, moderate, and high ligand expression groups was 20.5 months (95% CI, 13.8–27.2), 13.5 months (95% CI, 2.7–24.2), and 7.4 months (95% CI, 0.3–14.6), respectively. The difference between the low and high ligand expression groups was significant (*P* = 0.014).

To further understand the ICLs expressed on HEVs, we selected four important ligands—Gal-9, MHC II, Gal-3, and PD-L1, which were found to be significantly related to CD8^+^ T cell entry into the TLSs, and each of which was considered a risk indicator. In patients with a high expression of one or more of the four ligands, the risk level was higher. For patients with a high expression of 1–2 ligands, the risk level was I–II; and for those with a high expression of 3–4 ligands, the risk level was III–IV. For patients with a low expression of all four ligands, the risk level was zero. The survival of patients with different risk levels was studied. We observed that the median DFS of patients for the risk 0, I–II, and III–IV groups was 20.5 months (95% CI, 14.2–26.8), 13.5 months (95% CI, 5.0–21.9), and 7.2 months (95% CI, 3.5–14.6), respectively. The differences between the risk 0 and III–IV groups, and the risk 0 & I–II and III–IV groups were significant (*P* = 0.002, respectively; **Figure 6E**). Together, these data provide evidence that low ICL expression in mature HEVs is positively associated with better DFS compared with that associated with a high expression on these ligands.

To quantify the negative effect of ICLs of HEVs on survival, the ROC was analyzed to assess the accuracy of ICL scores in predicting survival (**Figure 6F**). Patients with a DFS shorter than the median (15.3 months) were considered to have survival loss, whereas patients in whom the DFS was longer than the median DFS were considered to have survival benefits. The data showed that among the five indices, only the ICL_total_ score had large AUC values (AUC = 0.705) with a *P*-value < 0.05 (*P* = 0.029), indicating that the ICL_total_ score had a strong prognostic predictive power. The cutoff value for the ICL_total_ score was evaluated, and a score of two was identified as the cutoff value that conferred 66.7% sensitivity and 66.7% specificity. Therefore, an ICL_total_ score > 2 predicted poor survival.

## 4 Discussion

**Figure 7:**
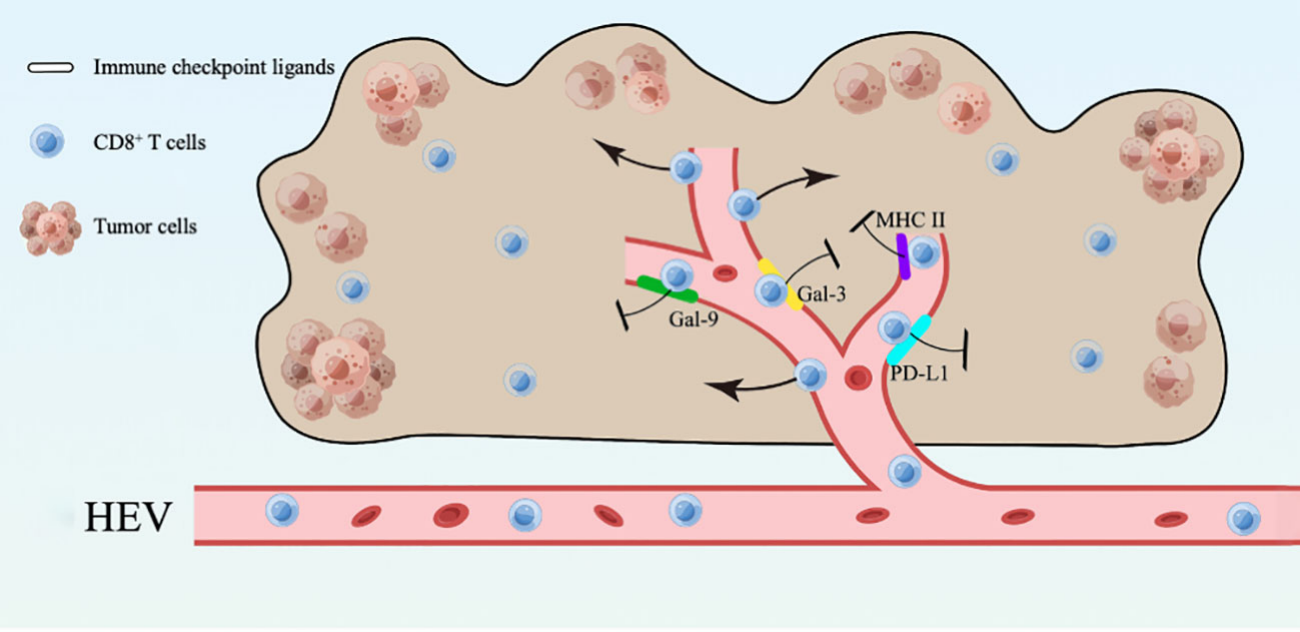
An overview of the present study.

In this study, we found that mature HEVs are participated in regulating the CD8^+^ T subsets delivery to the TLSs, but the ICLs expressed on mature HEVs (not blood vessels or lymphatics) are capable of affect CD8^+^ T lymphocytes infiltration negatively. Furthermore, the expression of ligands on HEVs may be an indicator of both CD8^+^ T cell infiltration and survival, according to our ICL score model, in which a high ICL_total_ score > 1 represent a weak CD8^+^ T cell infiltration and a high ICL_total_ score > 2 predicts poor survival. Novel foundings of the HEVs controlling lymphocyte delivery into the TLSs are presented in this study, along with a model of the total lymphocyte load (ICLtotal) score that is indicative of patient prognosis. Additionally, an overview was provided to illustrate the present study (**Figure 7**).

There have been considerable improvements in antitumor immunotherapy due to the immune checkpoint blockade (ICB). However, many patients fail or relapse after ICB treatment, indicating the limitations of antitumor immunotherapy (32, 33). Furthermore, the outcomes of ICB treatment are influenced by the quality and magnitude of the lymphocytes response within the TME (12). In cancer patients, prognosis and outcomes of therapeutic interventions are predicted by tumor-infiltrating lymphocyte levels (34, 35), and increased tumor-infiltrating lymphocyte infiltration may improve patient prognosis. Therefore, the entry of immune cells into the TME has attracted increasing attention. In tumors, lymphocytes are mainly extravasated through HEVs (4). Assia et al. (4) revealed that the maturity of HEVs plays a critical role in their molecular function. The maturity of HEV endothelial cells, in addition to their number, is critical for the lymphocyte delivery mediated by HEVs. Increasing the frequency and maturity of HEV endothelial cells can increase the outcomes and clinical response of ICB treatment. In our study, we uncovered the relationship between TLS maturity and CD8^+^ T cell infiltration: patients with mature TLSs had higher percentages of TLS-infiltrating CD8^+^ T, whereas patients with less mature or naïve TLSs had fewer CD8^+^ T in the TLSs, suggesting mature HEVs can positively affect CD8^+^ T cell delivery to the TLSs. Furthermore, the results of our previous study (24) demonstrated that patients with mature TLSs had a longer DFS. Taking our results together, we conclude that the maturity of HEVs/TLSs strongly affects tumor immunity and survival via regulating CD8^+^ T cell entry. However, the mechanism through which the maturity of HEVs/TLSs influences CD8^+^ T cell entry into tumors remains unknown, and the underlying molecular mechanisms remain poorly understood.

The results of the multiplex immunohistochemistry analyses in this study revealed that the checkpoint ligand expression on HEVs of mature TLSs is lower, which may be a key factor affecting CD8^+^ T cell entry into the TLS. There are immune checkpoints that regulate inhibitory or stimulating immune responses through ligand-receptor pairs (31). Immune cells, particularly the T cells express immune checkpoints and their ligands can be found in the TME (36). Ligand expression is essential for triggering signals via immune checkpoint receptors. Immune evasion is the primary function of tumor-associated immune checkpoints, and their suppressive functions mostly depend on ligand-induced signaling (37, 38). When engaged with their ligands, checkpoint signaling can be triggered (37); however, their activity can be easily stopped by preventing ligand-receptor engagement using blocking antibodies (36). Admittedly, a negative relationship was observed between the expression of checkpoint ligands on HEVs and the CD8^+^ T cell infiltration, this is mostly due to the decrease of CD8^+^ T cell infiltration, however, the inhibition of the proliferation and activation of CD8^+^ T cells caused by the expression of the ligands on the HEVs maybe another potential reason. Concerning the HEVs are major vessels that mediate lymphocyte trafficking, and few studies report the HEVs has the ability to affect the proliferation and activation of lymphocyte, we speculate that the HEVs with high expression of checkpoint ligands have a primary effect on negatively influencing the entry of CD8^+^ T cells into tumors, although they might also inhibit the CD8^+^ T cells proliferation at the same time. The deep mechanism that how ligands expressed on HEVs affect the CD8^+^ T cell infiltration is ongoing.

In recent years, many researchers have uncovered the sophisticated regulation of checkpoint-ligand engagement, where different ligands show distinct signaling mechanisms that impact antitumor immunity (37), the molecular functions of immune checkpoints and their ligands remain poorly understood. In this study, we selected TIM-3, LAG-3, and PD-1, the three most common immune checkpoints which are also critical for immune regulation, and analyzed the expression of their corresponding ligands in mature HEVs. Not every ligand was included in the analysis: a ligand of LAG-3, α-syn, was not observed because it is mainly involved in intercellular synaptic transmission and does not substantially participate in immune-related activities (39, 40). Another ligand not included was PD-L2, a PD-1 ligand. The functions of PD-L2 are more sophisticated than those of PD-L1, which is the primary ligand of PD-1; by binding to PD-1, PD-L2 also facilitates the inhibitory functionality of PD-1 (36). In addition, PD-L2 can engage another receptor, RgmB (41), which can activate T cells (41, 42). Considering the uncertainty regarding the function of PD-L2, we excluded PD-L2 in our study. Finally, we found that the ICLs expressed on mature HEVs negatively affect CD8^+^ T cell entry into the TLSs, which has not been previously reported. Regardless of the ligand expression at the exact checkpoint or the total ligand expression, we observed that lower TLS-infiltrating CD8^+^ T cell percentages were associated with higher ligand expression, the differences were statistically significant. Additionally, not every ligand was involved in the CD8^+^ T cell delivery to TLSs; only Gal-9, MHC II, GAL-3, and PD-L1 negatively regulated CD8^+^ T cell entry into tumors, which also demonstrates the complicated functions of ICLs. Additionally, from the results of survival analysis, we found that DFS was shorter for patients with high ICL expression on HEVs, whereas patients with low expression of these ligands had significantly better DFS. Until now, this is the first study to reveal an association between the expression of ICLs in HEVs and survival. Thus, the value of the ICLs expressed in HEVs was determined.

To quantify the influence of ICL expression on TLS-infiltrating CD8^+^ T cells and survival, we constructed an ICL score model and performed ROC curve analysis. The AUC of each ICL score was measured, we identified the cut-off value of “1”, where an ICL_total_ score greater than “1” predicts weak CD8^+^ T cell infiltration (*P* < 0.001). Moreover, another cutoff ICL_total_ score was calculated: an ICL_total_ score greater than “2” indicates shorter survival (*P* = 0.029). Based on the ROC curves and their cutoff values, we consider that the ICL_total_ score will help clinicians predict the ICB treatment response and partly predict the DFS of patients.

The present study has several limitations. Only three ICLs were investigated; other immune checkpoints such as TIGIT and CTLA-4 were not considered. Accordingly, further investigations are needed that include ligands of other immune checkpoints. Our study provides preliminary evidence supporting the importance of ICLs expressed on HEVs. Moreover, only 42 patients were recruited; relevant large-scale clinical research is ongoing.

In summary, our study highlights the importance of ICLs in mature HEVs. Mature HEVs (not blood vessels or lymphatics) can positively affect the infiltration of CD8^+^ T subsets. Additionally, the ICLs expression on mature HEVs may negatively regulate the delivery of CD8^+^ T population to TLSs. Besides, according to our ICL_total_ score model, the expression of ICLs on HEVs can predict patients’ survival, where a high ICL_total_ score predicts a poor prognosis.

## Data availability statement

The raw data supporting the conclusions of this article will be made available by the authors, without undue reservation.

## Ethics statement

The studies involving humans were approved by Human Investigation Committee of Tianjin Medical University Cancer Institute and Hospital. The studies were conducted in accordance with the local legislation and institutional requirements. Written informed consent for participation in this study was provided by the participants’ legal guardians/next of kin.

## Author contributions

XR: Project administration, Writing – review & editing. HZ: Supervision, Writing – review & editing. FW: Supervision, Writing – review & editing. JL: Formal analysis, Methodology, Writing – original draft. XS: Methodology, Writing – original draft. YL: Writing – original draft. JW: Writing – original draft. XY: Writing – original draft. HW: Writing – original draft. QS: Writing – original draft. ZH: Writing – original draft.
