# Supplementary material for: Immune checkpoint ligands expressed on mature high endothelial venules predict poor prognosis of NSCLC: have a relationship with CD8+ T lymphocytes infiltration
Source: Front Immunol. 2024 Feb 8;15:1302761. doi: 10.3389/fimmu.2024.1302761 (PMC10882939; doi:10.3389/fimmu.2024.1302761)
Supplement: Supplementary file 1 [file DataSheet_1.docx]

**Supplementary**

**Figure S1**

**
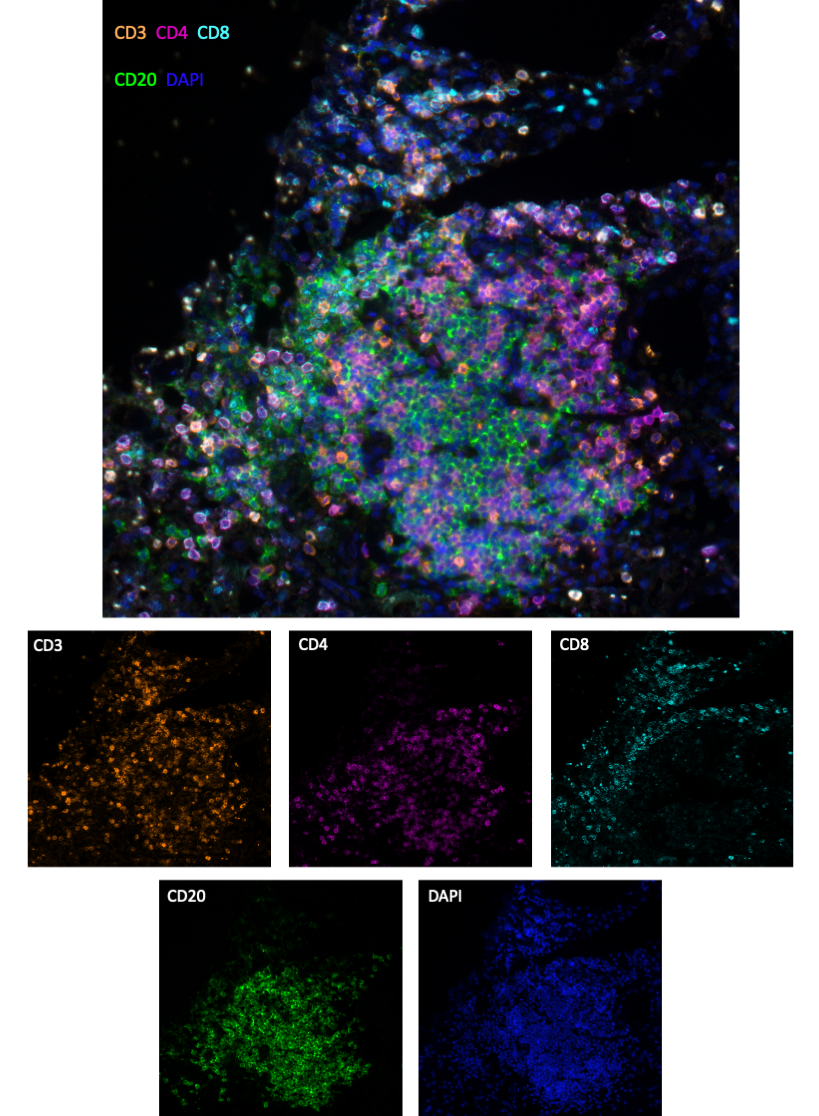
**

**Figure S1**. Represent image of CD3^+^, CD4^+^, and CD8^+^ T cells and the TLSs (represented by B cells).

**Figure S2**.


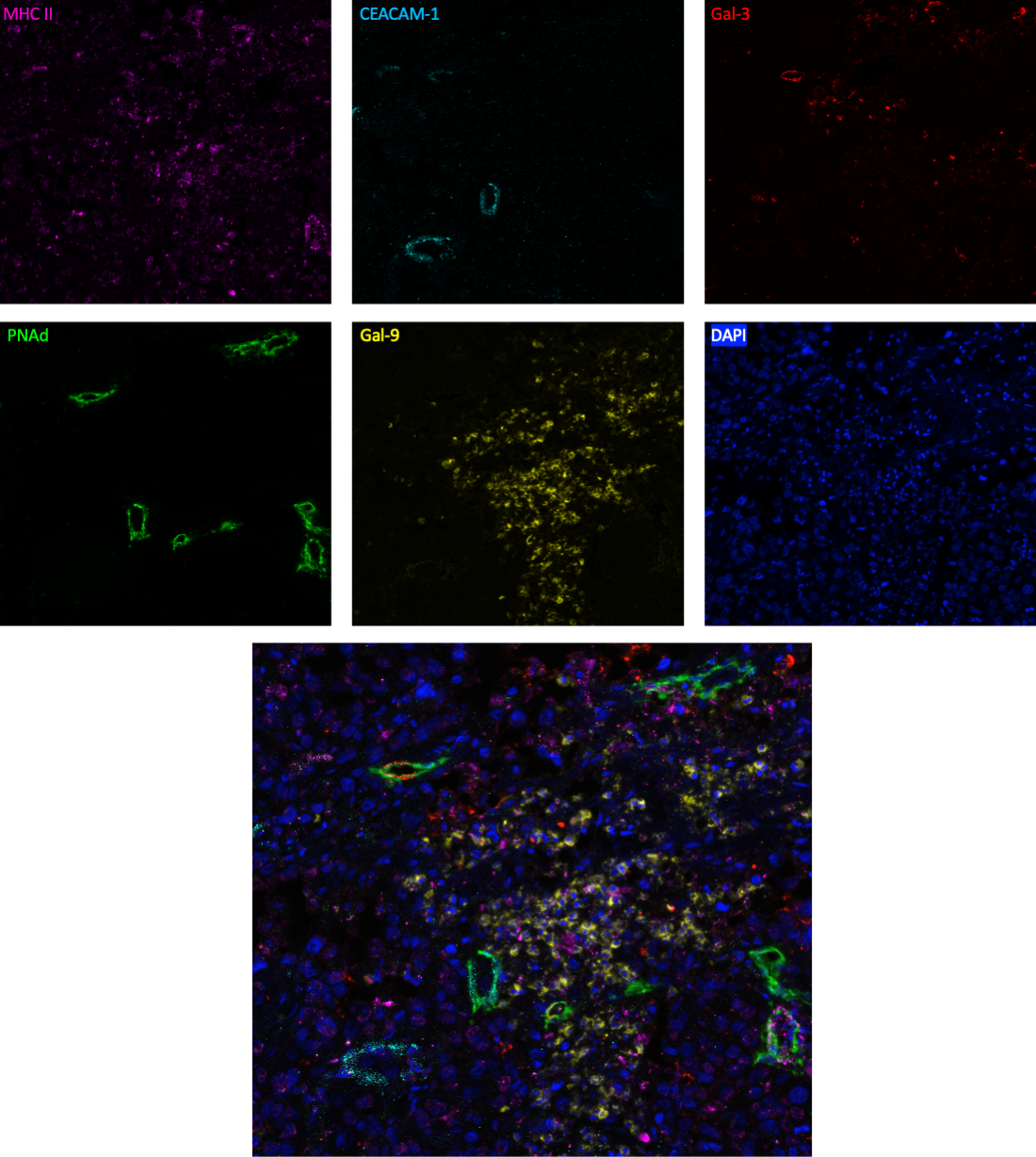


**Figure S2**. Representative multiplex immunohistochemistry staining image (20×) showing ligands of MHC II, CEACAM-1, Gal-9 and Gal-3 expressed on mature HEVs in single channel.

**Figure S3**

**
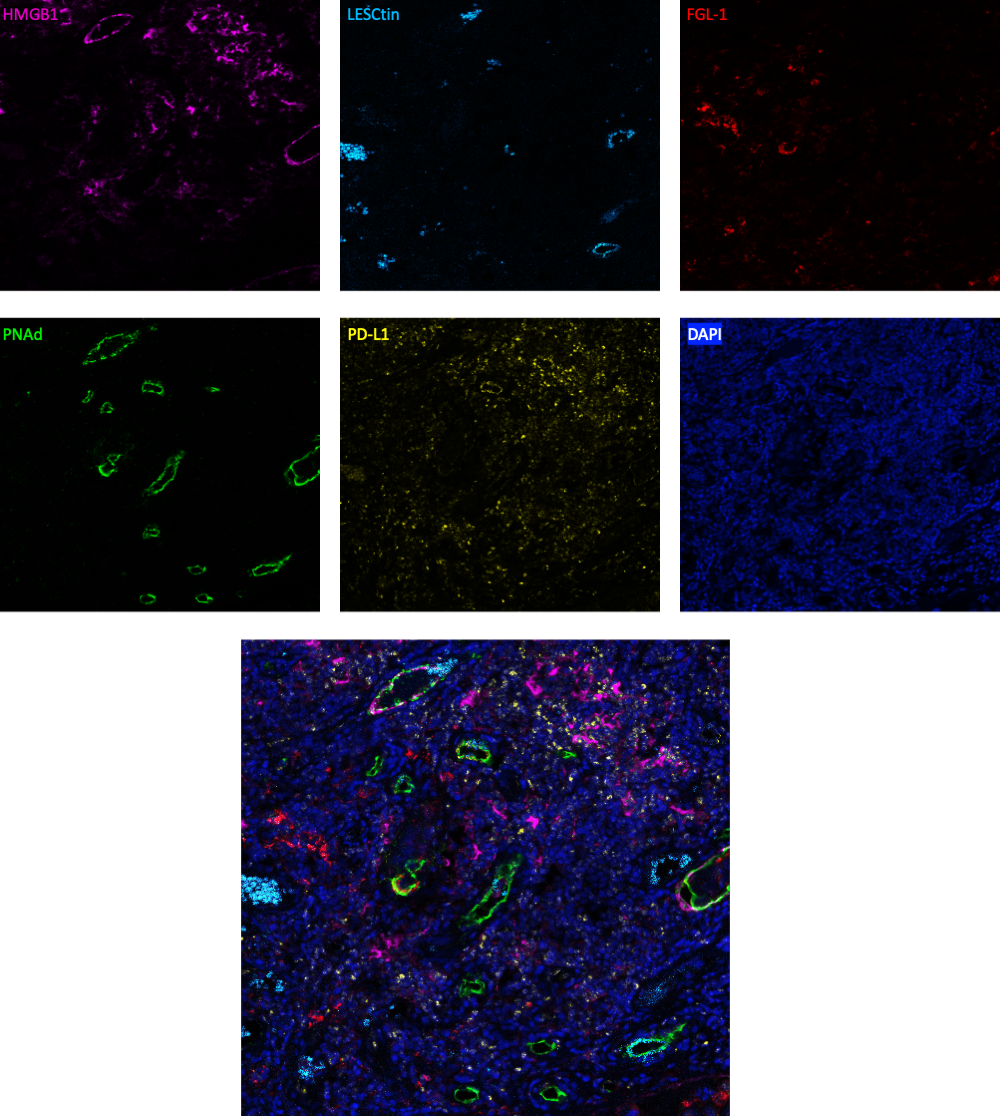
**

**Figure S3**. Representative multiplex immunohistochemistry staining image (20×) showing ligands of HMGB1, LSECtin, PD-L1 and FGL-1 expressed on mature HEVs in single channel.

**Figure S4**

**
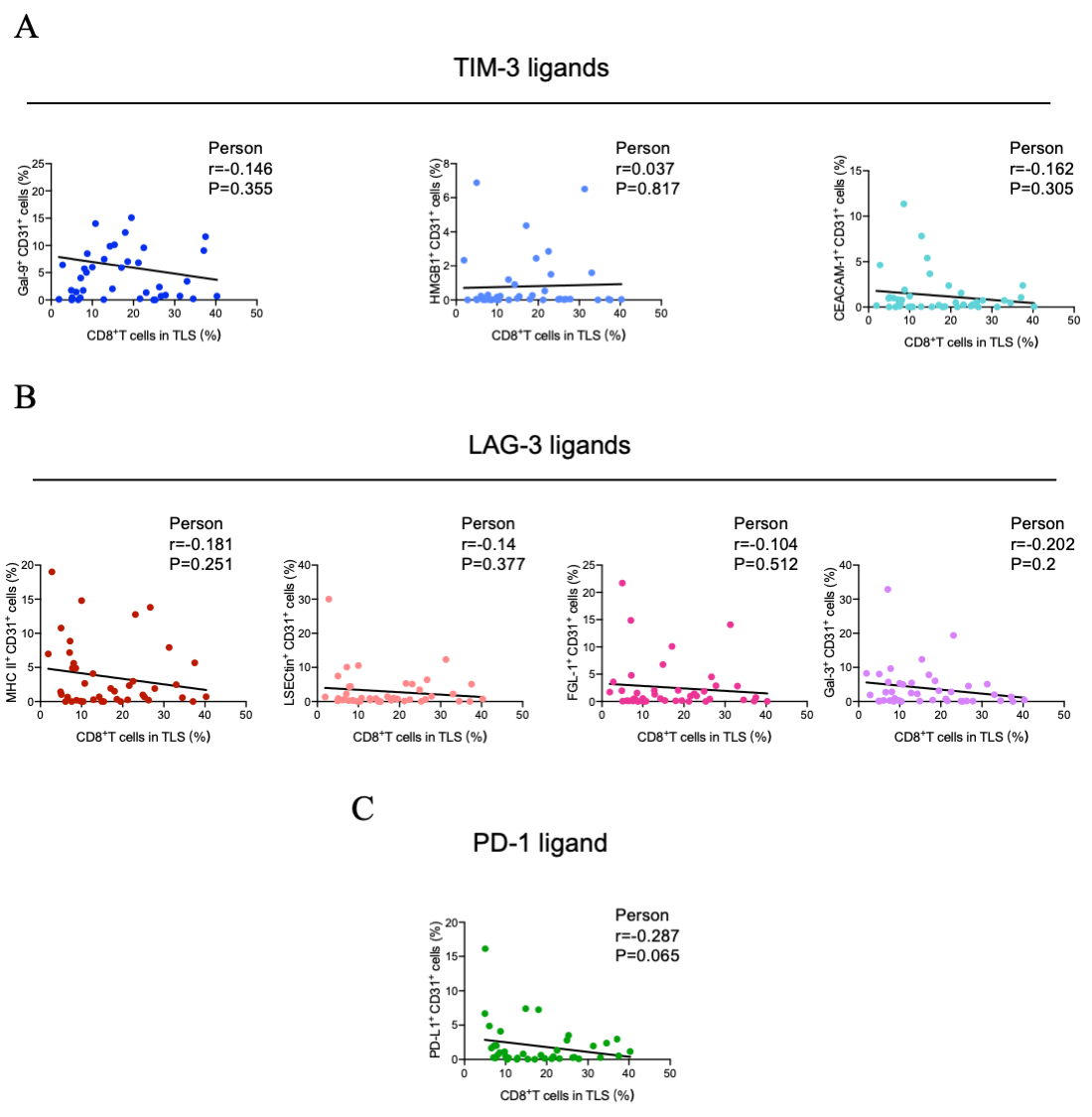
**

**Figure S4**. Correlation between **(A)** TIM-3, (**B**) LAG-3, and (**C**) PD-1 ligands expressed in blood vessels and TLS-infiltrating CD8^+^ T-cell frequency. HEVs, high endothelial venules. TLSs, tertiary lymphoid structures. Data are presented as mean ± SD. * *P* < 0.05; *** *P* < 0.01; ns, not significant according to unpaired two-tailed Student’s t-test.

**Figure S5**

**
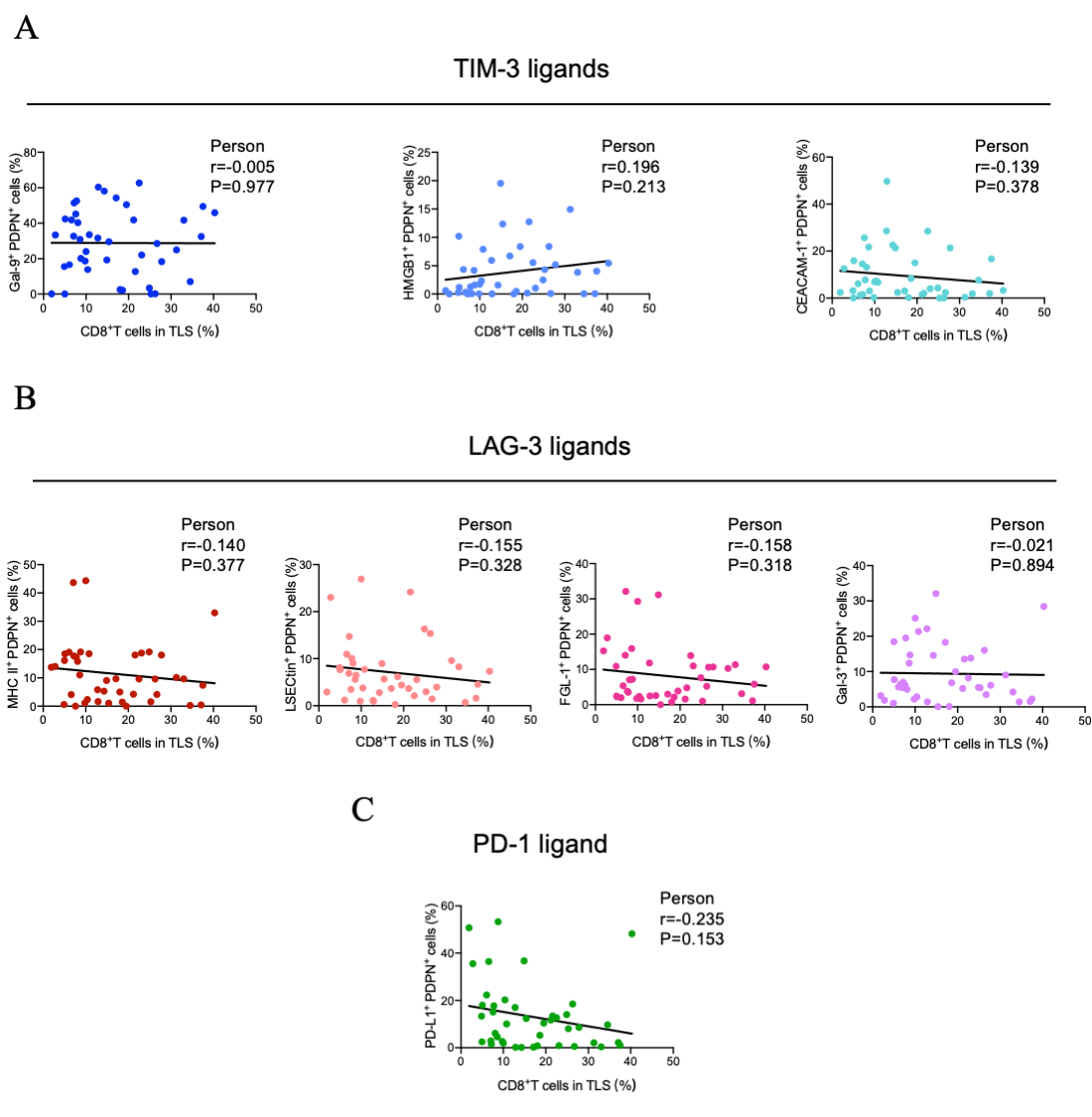
**

**Figure S5**. Correlation between **(A)** TIM-3, (**B**) LAG-3, and (**C**) PD-1 ligands expressed in lymphatics and TLS-infiltrating CD8^+^ T-cell frequency. HEVs, high endothelial venules. TLSs, tertiary lymphoid structures. Data are presented as mean ± SD. * *P* < 0.05; *** *P* < 0.01; ns, not significant according to unpaired two-tailed Student’s t-test.

**Figure S6**.


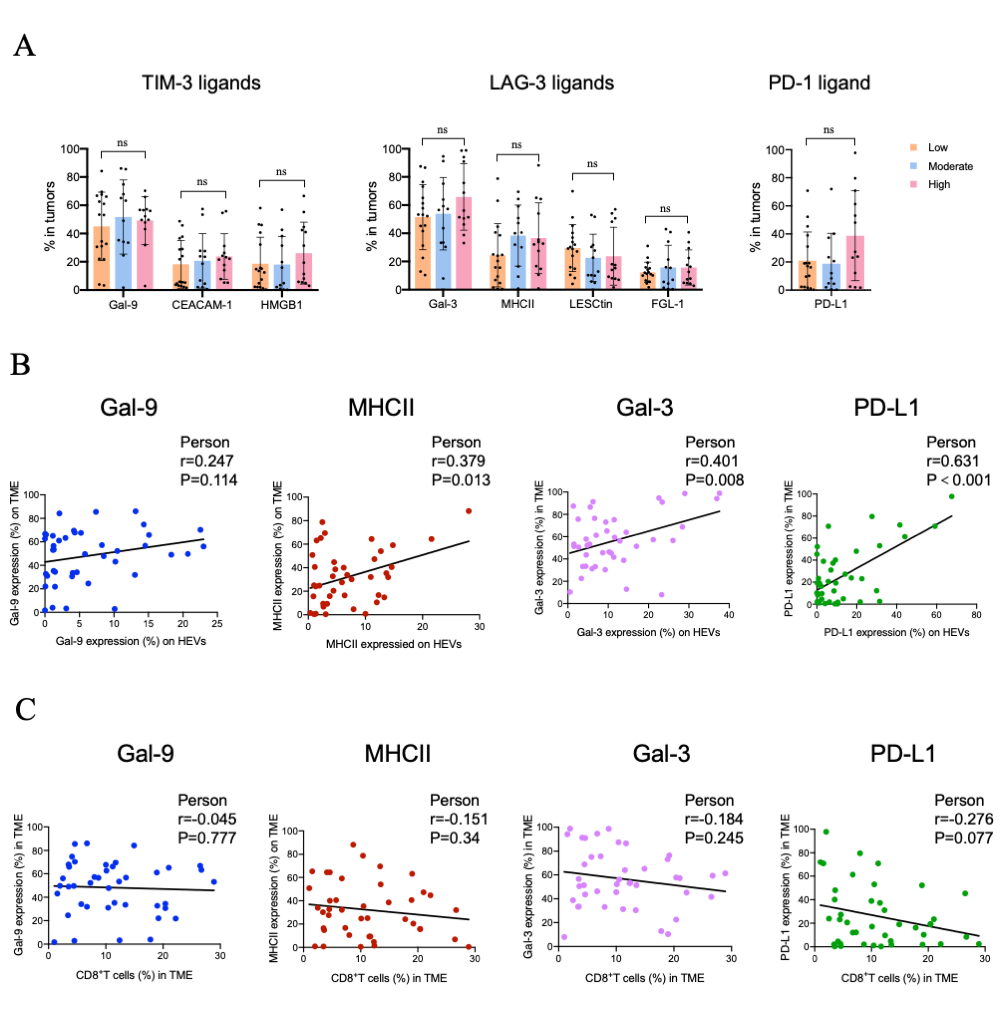


**Figure S6**. (A) Immune checkpoint ligands expression in TME of patients with different ICL expression levels. (B) Correlation between the 4 important ligands expressed in TME and expressed on mature HEVs. (C) Correlation between the 4 important ligands expressed in TME and tumor-infiltrating CD8^+^ T cell frequency.

**Figure S7**

**
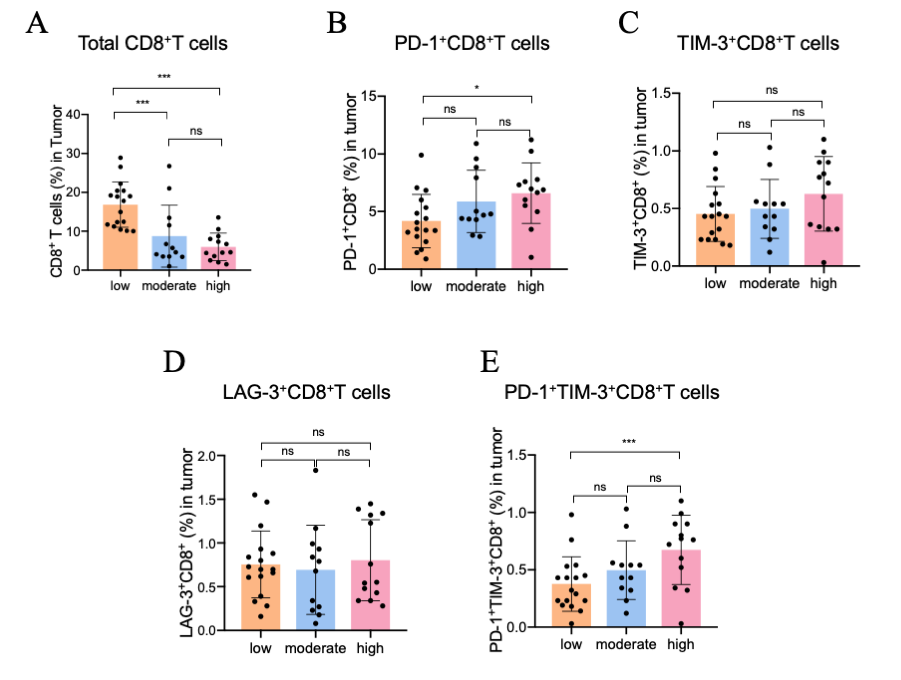
**

**Figure S7**. Percentages of total tumor-infiltrating CD8^+^T cells (A) and the exhausted CD8^+^T cells (B-E) of patients with different ICL levels.
